# Characterization of the bovine type I IFN locus: rearrangements, expansions, and novel subfamilies

**DOI:** 10.1186/1471-2164-10-187

**Published:** 2009-04-24

**Authors:** Angela M Walker, R Michael Roberts

**Affiliations:** 1 Department of Veterinary Pathobiology, University of Missouri, Columbia, MO 65211, USA; 2 Department of Animal Sciences, University of Missouri, Columbia MO 65211, USA; 3 Christopher S Bond Life Sciences Center, University of Missouri, Columbia, MO 65211, USA

## Abstract

**Background:**

The Type I interferons (IFN) have major roles in the innate immune response to viruses, a function that is believed to have led to expansion in the number and complexity of their genes, although these genes have remained confined to single chromosomal region in all mammals so far examined. *IFNB *and *IFNE *define the limits of the locus, with all other Type I IFN genes except *IFNK *distributed between these boundaries, strongly suggesting that the locus has broadened as IFN genes duplicated and then evolved into a series of distinct families.

**Results:**

The Type I IFN locus in *Bos taurus *has undergone significant rearrangement and expansion compared to mouse and human, however, with the constituent genes separated into two sub-loci separated by >700 kb. The *IFNW *family is greatly expanded, comprising 24 potentially functional genes and at least 8 pseudogenes. The *IFNB *(n = 6), represented in human and mouse by one copy, are also present as multiple copies in *Bos taurus*. The *IFNT*, which encode a non-virally inducible, ruminant-specific IFN secreted by the pre-implantation conceptus, are represented by three genes and two pseudogenes. The latter have sequences intermediate between *IFNT *and *IFNW*. A new Type I IFN family (*IFNX*) of four members, one of which is a pseudogene, appears to have diverged from the *IFNA *lineage at least 83 million years ago, but is absent in all other sequenced genomes with the possible exception of the horse, a non-ruminant herbivore.

**Conclusion:**

In summary, we have provided the first comprehensive annotation of the Type I IFN locus in *Bos taurus*, thereby providing an insight into the functional evolution of the Type I IFN in ruminants. The diversity and global spread of the ruminant species may have required an expansion of the Type I IFN locus and its constituent genes to provide broad anti-viral protection required for foraging and foregut fermentation.

## Background

Viruses are constantly evolving to find more effective means to survive and multiply in their host species [1-3]. The immune defense system, in turn, exists in a perpetual state of co-evolution with the pathogens to limit infectious disease, a circumstance often likened to an "arms race." The primary defense mechanism against viruses in vertebrates is Type I IFN (interferon) of the innate immune system [4]. It can reasonably be argued that complex organisms like mammals can only survive as long as immune defenses can adjust to the strategies of invading pathogens. Accordingly, a rapidly evolving, adaptable IFN system is essential to mammals if they are to endure viral infections. Type I IFN are also pleiotropic cytokines, with significant roles in modulating adaptive immunity, cell proliferation and cell death, and numerous other processes vital to mammalian health and survival [1]. Most likely as a response to these challenges, Type I IFN demonstrate a complex evolutionary history that has resulted in the divergence of at least eight distinct subfamilies: IFN-kappa (IFNK), IFN-beta (IFNB), IFN-epsilon (IFNE), IFN-delta (IFND), IFN-zeta (IFNZ), IFN-alpha (IFNA), IFN-omega (IFNW), and IFN-tau (IFNT) [5].

Mammalian Type I IFN probably emerged during tetrapod evolution from an older cytokine family, Type III IFN, which provides the primary viral defense mechanism in fish [6,7]. It is difficult to determine exactly when Type I and Type III IFN diverged because no Type I IFN has been identified in amphibians, but the split definitely occurred prior to the divergence of birds and mammals approximately 310 million years ago (MYA) [5,8]. Type III IFN, known more commonly in mammals as either IFN-lambda (IFNL) or interleukin (IL)28 and IL29, is encoded by a five exon gene, opposed to the single exon Type I IFN, and acts through a different receptor complex than Type I IFN [9,10]. Despite these differences, both Type I and Type III IFN have similar mechanisms of induction, activate the same signaling pathways, and trigger the same biological actions in the target cell [11]. Type III IFN has been retained in some mammalian species including humans and mice but has been lost in others [12]. Even when present, it appears to have assumed a less dominant role as an antiviral agent [11] and may have been supplanted as major player in antiviral defense with the emergence of contemporary Type I IFN.

All Type I IFN elicit an antiviral response, but some may play a more dominant role as first responders than others. IFNA and IFNB were the first Type I IFN to be characterized in human and have been assumed to constitute and the primary viral defense mechanism [13,14]. IFNA is released by almost all cell types and a few of its family members, specifically human IFNA2a and IFN2b, are currently approved for treatment of a range of viral diseases including hepatitis B and C, condylomata acuminate (genital warts), and AIDS-related Kaposi sarcoma [15]. IFNB is the main IFN secreted by fibroblasts in response to a viral challenge, but is clearly produced by multiple cell types [16]. It acts in the immediate antiviral response and helps regulate the later expression of several *IFNA *[17]. IFNW and IFNZ both appear to have developed specific niches in antiviral protection for certain species. IFNW has been implicated in protection against specific viruses, such as parvovirus, particularly in cats [18,19], while murine IFNZ provides a unique combination of high antiviral activity with relatively low lymphomyeolosuppresive activity [20], suggesting it may act to suppress viruses targeting the bone marrow and spleen. IFNK is predominately expressed in keratinocytes where it is acts through a unique cell-associated viral protection mechanism [21,22]. IFNE is expressed in a variety of cell types, but has been suggested on the basis of rather meager evidence to serve a specific role in reproductive tissues either in viral protection or early placental development [5,23]. IFND and IFNT, on the other hand, are not induced by viruses but instead are released by the early pre-implantation embryos of swine and ruminant species, respectively, where they appear to trigger responses in maternal uterine endometrium that allow the pregnancy to become established [24,25].

The arrangement of Type I IFN genes within the locus likely reflects the origins and subsequent evolution of individual family members. All Type I *IFN *in human and mouse are clustered in an approximately 400 kb length of DNA, located on the short arm of chromosome 9 (9p21) in human and on the centromere-proximal region of chromosome 4 (4C4) in mouse [26-28]. Two genes of ancient origin, *IFNB *and *IFNE*, define the outer limits of the locus. All the other Type I *IFN *genes, except *IFNK*, are distributed between these two ancient genes, indicating the locus has expanded internally as IFN genes duplicated and then evolved into their respective families [27]. However, species-specific expansion and contraction of families has occurred, with some IFN families only existing in certain taxonomic groups. For example, *IFND *has only been identified in the pig and is absent in the mouse and human, while *IFNZ *is represented in the mouse, but only remnants of the gene has been found in rats, while it is completely absent in humans [20,25,29]. The *IFNW*, which are considered to have arisen from the *IFNA *at least 129 MYA [16,30], constitute a particularly variable grouping. A single functional *IFNW *and at least two pseudogenes are present in humans, but only a single pseudogene can be identified in mice [27]. Even more bewildering, the family appears to have expanded in cats, which, on the basis of cDNA evidence, possess at least 10 variants [31], but not even a relic of the open reading frame can be found in the related carnivore, the dog [32]. Ruminant species, such as cattle, are known to possess several, apparently functional, *IFNW *[33,34]. There is also one example of a Type I family, the *IFNT*, that arose relatively recently (36 MYA) in the lineage to the ruminant artiodactyls. As a consequence, the *IFNT *are absent from all species except those in the sub-order Ruminantia [33,35]. Together, these data suggest that novel *IFN *genes can be gained and existing genes discarded in response to specific environmental challenges, which most likely include threats from emerging new pathogens. In addition, existing *IFN *may become co-opted into new roles unrelated to viral pathogenesis, as has occurred in the case of the IFND [20,25,29].

Although it has been clear for some time that there are similarities in the organization of the Type I IFN locus of cattle and that of other species [36,37], it was equally evident that the bovine locus must have some unique features, most notably because of the existence of the *IFNT*, genes unique to ruminant species whose protein products, although active in antiviral assays, have a primary role as hormones of pregnancy [38]. Cattle also have multiple *IFNB *while all non-ruminant species so far examined possess only a single copy *IFNB *[5]. Together these findings suggest either a decreased restriction on duplication of Type I IFN genes in cattle or evolutionary pressure to acquire additional genes. The recent sequencing of the bovine genome has provided the first opportunity for a detailed study of the Type I *IFN *locus in a ruminant species. Here we provide a detailed description and full annotation of the bovine locus and some inferences about its evolutionary history.

## Methods

### Annotation

Most of the *IFN *gene candidates were identified through the National Center for Biotechnology and Information (NCBI)'s bovine genome resource by using the basic local alignment search tools (BLAST) [39]. Additional searches were performed through NCBI by using the appropriate genome resource [40] for other species, which are discussed later in this section, and by using the basic nucleotide BLAST suite [41,42]. Several combinations of BLAST algorithms and databases within NCBI were utilized for this work and are described below [43].

### BLAST algorithms

1. MegaBLAST was designed to compare highly related nucleotide sequences and works best when the target sequence has a 95% identity or higher to the query sequence.

2. Cross-species megaBLAST, also referred to as discontiguous BLAST, is a derivative of megBLAST that ignores certain bases, thereby allowing mismatches. It was designed to compare nucleotide sequences from one species to nucleotide sequences in another species.

3. BLASTN also compares nucleotide query sequences to a nucleotide database. This algorithm is slower than megaBLAST, but it can identify shorter sequence matches than megaBLAST. It was not specifically designed for cross-species comparisons.

4. TBLASTN was designed to compare a protein sequence with a nucleotide database dynamically translated in all reading frames.

### NCBI databases

1. The "genome (reference)" database represents the most current publicly available assembly of a genome. The most current assembly of the bovine genome at the time this work was completed was assembly 3.1. The most current assembly for other species examined in this work are placed in parenthesis here – human (36.2), mouse (37.1), horse (1.1), and dog (2.1).

2. The "WGS contigs" database contains the contigs, or overlapping unassembled sequences, that forms the basis for the assembled genome. Both pig and cat do not have an assembled genome available at this time and only the "WGS contigs" database could be searched for genomic information for these species.

3. The "traces-WGS" database contains the trace data for whole genome shotgun sequence (WGS) bacterial artificial chromosome (BAC) end sequencing. This database contains single pass sequencing reads that are not trimmed based on quality or vector contamination.

4. The "nucleotide collection (nr/nt)" database contains all Genbank, RefSeq, EMBL (Europe's primary nucleotide database), DNA Database of Japan (DDJB), and many Protein Databank (PDB) sequences. The "nucleotide (nr/nt)" database is subdivided into "human nucleotide (nr/nt)," "mouse nucleotide (nr/nt)," and "others nucleotide (nr/nt)" databases. The "others nucleotide (nr/nt)" database does not contain any mouse or human sequences.

Bovine *IFNB*, *IFNA*, *IFNW*, and *IFNT *cDNA sequences (Table 1) from GenBank were used to perform a megaBLAST search in the bovine "genome (resource)" database. Human *IFNE*, murine *IFNZ*, porcine *IFND*, human *IFNK*, and human *IFNL/IL28-29 *sequences (Table 1) were queried with cross-species megaBLAST in the bovine "genome (reference)" database because no bovine homologues for the latter group of genes have been reported. The translated sequence for each of the non-bovine *IFN *cDNA were also queried with TBLASTN in the bovine "genome (reference)" database because the TBLASTN algorithm can often identify homologues that are not detected through other searches. *IFNL/IL28-29 *nucleotide and amino acid sequence were also queried in the bovine "WGS contig" database by using a cross-species megaBLAST and TBLASTN search, respectively, to verify no sequence with high identity to *IFNL/IL28-29 *in the bovine genome was missed. Specific genes were analyzed in the bovine "traces-WGS" database to verify frameshift mutations or nucleotide variations from the query sequences.

**Table 1:** Query sequences used for the genomic searches.

**Species**	**Gene**	**Accession No.**	**Species**	**Gene**	**Accession No.**
Bovine	IFNA	AY325272	Bovine	IFNT	AF196324
Bovine	IFNA	M10954	Porcine	IFND	Z22707
Bovine	IFNA	AY523531	Porcine	IFND	Z22706
Bovine	IFNA	DQ396807	Human	IFNK	NM_020124
Bovine	IFNA	Z46508	Human	IFNE	NM_176891
Bovine	IFNB	M15478	Human	IFNL	AY184374
Bovine	IFNW	M11002	Human	IFNL	AY184373
Bovine	IFNT	M31557	Human	IFNL	AY184372
Bovine	IFNT	AF196320	Murine	IFNZ	NM_197889
Bovine	IFNT	AF196322			

The Bovine Genome Sequencing and Annotation Consortium created a consensus predicted gene set through an algorithm, termed GLEAN, developed during the annotation of the honey bee that used latent class analysis to automatically combine disparate gene prediction evidence [44]. Since the majority of positive megaBLAST, cross-species megaBLAST, and TBLASTN matches were clustered on two scaffolds, Chr8.25 [Genbank: NW_001495421] and Chr8.34 [Genbank:NW_001495430], all GLEAN models on those two scaffolds were also annotated through Apollo [45,46]. In brief, Apollo is a genome annotation viewer and editor that was originally designed for the annotation of the *Drosophila melanogaster *genome. The Bovine Genome Sequencing and Annotation Consortium created input files for Apollo containing EST matches, cDNA matches, translated protein matches, and gene model data including all GLEAN models for the bovine genome assembly 3.1. GLEAN models present on scaffolds Chr8.25 and Chr8.34 that had not been identified in the aforementioned searches were queried through BLASTN and discontiguous megaBLAST in the "others nucleotide collection (nr/nt)" and "human nucleotide collection (nr/nt)" databases to verify their status as IFN genes or another gene family. Discontiguous megaBLAST and TBLASTN searches in human, mouse, equine, porcine, feline, and canine "genomic (reference)" and "WGS contigs" databases were performed for the unique IFN family discovered during the annotation of Chr8.34.

The 64 identified IFN genes and pseudogenes and the original query cDNA from Genbank (Table 1) were aligned through CLUSTALW in BioEdit version 7.09 [47,48]. A pairwise comparison to known *IFN *nucleotide sequences was performed through the Maximum Composite Likelihood method in MEGA version 4 (MEGA4) [49] to determine the IFN family for each gene [50,51].

*IFNT *was queried with megaBLAST in the bovine "traces-WGS" database to validate the number of IFN genes present in the genome. Bovine sequence matches that had greater than 94% sequence identities to the query *IFNT *for more than 400 basepairs (bp) were visually inspected. An *IFNT *match was counted as a positive if the sequence had greater than 98% identity to an *IFNT *cDNA in the portion of the trace with a quality score, available through NCBI, higher than 40 on a scale between 0 and 100. The total number of *IFNT *matches in the WGS contig database was divided by the bovine genome coverage to approximate the total *IFNT *gene number.

### Phylogenetic Reconstruction

Alignments for the genomic *IFN *ORFs were created through ClustalW in BioEdit version 7.09, with individual genes denoted by their GLEAN numbers. Phylogenetic trees were constructed in MEGA4 through the Neighbor-joining (NJ) method with bootstrapping test (1000 replicates). The tree was rooted to IFNK [5] and a second tree was created with the assumption of a non-uniform rate of change between sites (gamma = 1).

### Identification of repetitive elements

The localization and identity of all repetitive elements were determined by using the RepeatMasker program [52], which uses the RepBase library of repeat elements [53]. Sub-locus 1, corresponding to 20000–711500 bp in scaffold Chr8.25, and sub-locus 2, corresponding to 2000–446000 bp in scaffold Chr8.34, sequences were first selected through Apollo and imported into a word processing program, Microsoft Word. All gaps within the scaffolds, which are represented by an "N" in the bovine assembly, were removed manually. IFN sub-loci sequences were then analyzed in RepeatMasker version 3.1.9 run in default mode with blastp version 2.0MP-WashU [54] to determine the percentage of repetitive elements. *Bos taurus *was set as the assumed species within the program parameters. Simple repeats and low complexity regions were not masked, which means they were not excluded as start sites for a BLAST match, and the matrix was optimized for 42% GC content based on sub-loci optimization pre-runs.

## Results and discussion

### IFN Gene Families in Bos taurus

Evidence for the presence of all previously known Type I IFN subfamilies except *IFNZ *was found on chromosome 8 of the bovine genome assembly 3.1 (Table 2). *IFNZ *has only been reported in mouse [27,55], and so its absence in *Bos taurus *was anticipated. Both *IFNK *and *IFNE *are present as single genes with intact ORF and are assumed to be functional, providing the first evidence that either of these subfamilies is present in ruminants. Bovine *IFNK *and *IFNE *have 81.2% and 84.7% nucleotide identity respectively when compared to their human orthologues, values that are similar to their degree of conservation between human, cat, dog, and pig (A. Walker, unpublished data). The *IFNW *family is greatly expanded compared to other species that have so far been examined. There are 24 potential *IFNW *and at least 8 pseudogenes. The query *IFNW *[Genbank:M11002] [34] exactly matches two *IFNW *annotated from the genome database (GLEAN 09983 [Bovine Genome Database temporary ID:2733] and GLEAN 10004 [Bovine Genome Database temporary ID: 2525]). The remaining *IFNW *range in sequence identity from 86% to 96% relative to the query sequence.

**Table 2:** Cross-species comparison of IFN subfamilies.

	**Gene Number**		
	
**Subfamily**	**Human**	**Mouse**	**Cow**
IFNK	1	1	1
IFNE	1	1	1
IFNB	1	1	6
IFND	0	0	0
IFNZ	0	2	0
IFNA	13	14	13
IFNW	1	0	24
IFNT	0	0	3
IFNX	0	0	3
IFNL	3	3	0

The number of predicted IFN genes in each subfamily based on genomic analysis of the mouse, human, and bovine are shown. Predicted pseudogenes based on frameshift mutations or stop codons within the first 100 aa of the coding sequence have been excluded from the table. The bovine Type I IFN locus has an expansion of both the IFNB and IFNW subfamilies. Cattle have also acquired two novel IFN subfamilies, IFNT and a previously unidentified IFN family, termed IFNX, discovered during this analysis.

The *IFNA *and *IFNB *are also present in multiple copies, with 13 and 6 genes, respectively, although neither family is as large as the *IFNW*. An apparent *IFNB *pseudogene, deemed nonfunctional due to a frameshift deletion, also exists.

*IFND *are only represented as three pseudogenes, a not unexpected finding, as a functional gene has only been reported previously for the pig [56].

Three apparently functional *IFNT *are found within the locus. Surprisingly none of these provide an exact match for any of the many cDNA and gene sequences that have previously been reported. Previous mRNA sequencing of the *IFNT *family had indicated that at least 18 bovine *IFNT *might exist [57]. Only three *IFNT *are present in the bovine genome assembly 3.1, however. One particular, well established sub-family, the *IFNT2 *grouping [57], is not represented at all in the assembly. Additional analysis revealed 45 acceptable matches to *IFNT *in the WGS contig database. Since the bovine genome at this time has 7.1 X coverage, the number of *IFNT *matches divided by this coverage value suggests the possibility of around six *IFNT*. One explanation is that these "extra" genes have been lost in the assembly process, but even this higher value is still significantly lower than the 10 to 18 *IFNT *previously believed to exist. Some of the latter are most likely alleles.

Most interestingly, we detected a novel Type I IFN, which, as we shall discuss later, consists of three potentially functional genes and one pseudogene, none of which provides a close sequence match with any previously described Type I *IFN*. For convenience, and until an appropriate nomenclature is approved, this new family will be termed *IFNX*.

A weak sequence identity to *IFNL *was found on chromosome 13, specifically located on scaffold Chr13.80 [Genbank:NW_001493172] from 635,850 to 636,120 bp. This sequence appears not to encode a functional gene in either the 3.1 assembly or the WGS contig database. These data suggest that the Type III IFN family exists only as a relic and is no longer a functional component in bovine pathogen defenses.

### Locus Map

**Figure 1:**
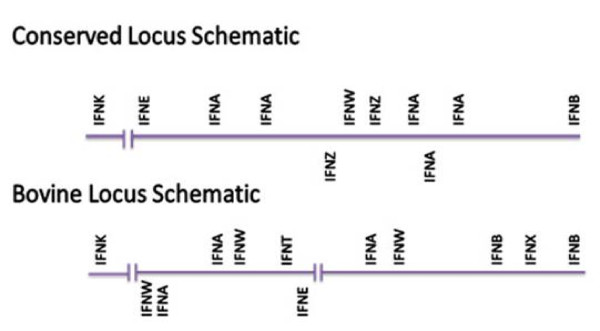
**Type I IFN locus schematics**. These schematics, while not drawn to scale, illustrate the basic characteristics of the locus in mouse and human, the Conserved Locus Schematic, and the bovine locus. Each *IFN *gene is represented by its abbreviated name and its position above and below the schematic line represents the direction of its transcription.

The Type I IFN locus is organized similarly in mouse and human, and possibly also in pig, i.e. there is relatively conserved synteny across rodents, primates, and swine. Two mammalian IFN genes of ancient origin, *IFNB *and *IFNE*, define the outer limits of the locus, with all the other genes, except *IFNK*, distributed between these two markers. The genes are predominantly (but not exclusively) localized on one strand and transcribed in the same direction as the *IFNB *and *IFNE *[27]. The relative arrangements of the murine and human *versus *the bovine *IFN *locus are illustrated in Fig. 1. The bovine Type I IFN locus is clearly organized differently than that of the other two species. Instead of a single stretch of DNA defining the locus, cattle have two sub-loci (1 & 2) encompassing 701 kb and 441 kb, respectively, separated by a gap estimated to be approximately 11 megabases (Mb) in assembly 3.1 (but ~750 kb in assembly 4.0). The gene density based on the both predicted open reading frames (Gene Sequence map) and bovine EST and mRNA alignments with the assembled sequence (BT UniG map) is lower in the region between the two IFN sub-loci than much of the rest of chromosome 8, but many genes are present and actively transcribed.

**Figure 2:**
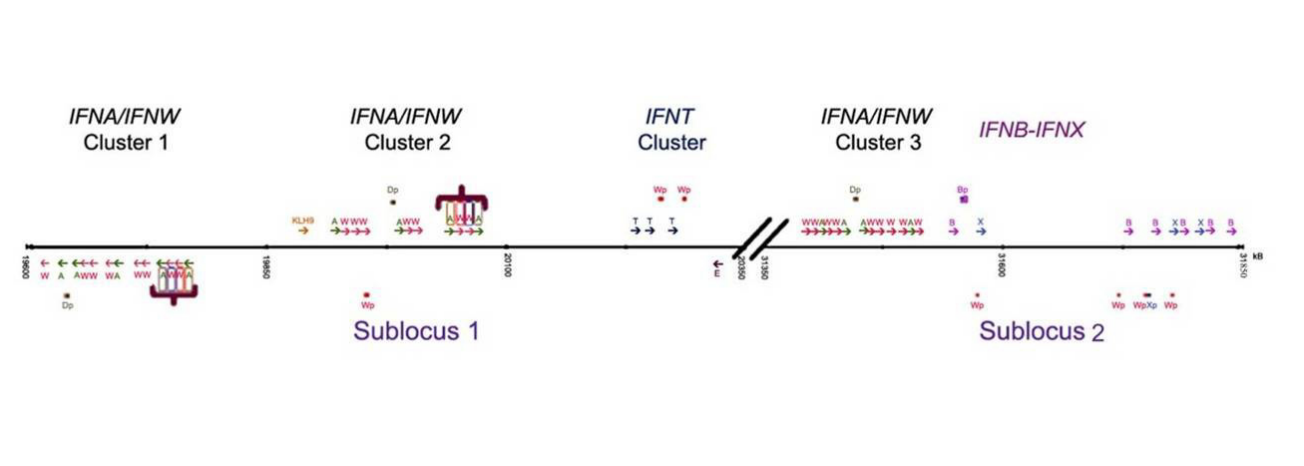
**Genomic map of the bovine Type I IFN locus**. Blast searches of the bovine genome database revealed that all matches to known *IFN *genes, except *IFNK*, reside within two sub-loci, illustrated in the gene map shown in Fig. 2. Both the position of each gene relative to the line and the direction of the arrow on the map denote the direction of transcription. The subfamily for each gene is designated by the final letter of the abbreviated name. Pseudogenes are indicated by a box instead of an arrow and the letter "p" after the subfamily designation. Specific gene clusters have been labeled according to the subfamily or subfamilies they contain. A recent example of gene duplication is illustrated in the *IFNW/IFNA *cluster 1 and 2. The bracketed gene set in *IFNA/IFNW *cluster 1 is a palindrome to the bracketed gene set in the *IFNA/IFNW *cluster 2 with identical coding sequences for genes specified by correspondingly colored boxes.

The following explanation of the locus organization has been based on the chromosome map assigned during the assembly process and reported by NCBI (Fig. 2). An *IFNB *defines the distal end (relative to the start of the chromosome map) of sub-locus 2 (Fig. 1 &2). *IFNE*, while present in the bovine, is located towards the distal end of sub-locus 1. The majority of the genes in both sub-loci are transcribed in the same direction as the distally placed *IFNB*, except one cluster of *IFNW *and *IFNA *and the solitary *IFNE*, which are transcribed in the opposite direction. *IFNK *is present in a single "copy" nearer to the start of the chromosome map and well separated (6.044 Mb) from the closest sub-locus (sub-locus 1) (Fig. 1). The bovine *IFNK *location is very similar to that in the human where a single *IFNK *is located 6.5 Mb from the Type I IFN locus [22] (Fig. 1).

There are three clusters of *IFNA/IFNW*. Two of them are on sub-locus 1, one at the proximal end, the second placed about half way along (Fig. 2). A gene set in the first *IFNA/IFNW *cluster 1 is a palindrome to one in the second cluster. The corresponding gene pairs have complete nucleotide identity within their coding regions, suggesting that the duplication or gene conversion event that led to their formation occurred quite recently. The third cluster of *IFNA/IFNW *is at the distal end of sub-locus 2, but lacks the duplicated group of four genes in *IFNA/IFNW *clusters 1 and 2.

Only one non-IFN gene is detectable within sub-loci 1 and 2, an intronless *kelch-like 9 *(*KLHL9*) located 33.5 kb proximal to *IFNA/IFNW *cluster 2 in sub-locus 1 (Fig. 2). The orthologous *KLHL9 *gene can be found in the Type I IFN locus of the mouse approximately 25 kb from the nearest functional *IFN *(*IFNA8*) [27] and 29 kb from the nearest *IFN *(*IFNA6*) in human. The fact *KLHL9 *has resisted duplication despite residing close to genes undergoing multiple duplications is noteworthy and possibly indicates that multiple copies of this gene are not well tolerated.

The presence of *KLHL9*, which appears to be under different evolutionary constraints than *IFNW *and *IFNA*, close to *IFNA/IFNW *cluster 2 suggested the cluster as whole might be under different evolutionary control than cluster 1 or 3. Evolutionary divergence rates do not indicate that this is the case, however (Table 3). While cluster 1 does have a slightly higher divergence rate than cluster 2, all three clusters are relatively constant in their rate of change. Inter-cluster divergence is actually very low in all three of the *IFNA/IFNW *clusters.

**Table 3:** Divergence within IFNA/IFNW clusters.

	IFNA/IFNW Clusters		
	
Subfamily	1	2	3
IFNW	0.086 ± 0.007	0.071 ± 0.007	0.063 ± 0.006
IFNA	0.053 ± 0.006	0.04 ± 0.006	0.045 ± 0.006

Divergence rate ± standard error for *IFNA *and *IFNW *within clusters was calculated by using the Maximum Composite Likelihood method. The number of base substitutions per site from averaging over all sequence pairs within each cluster is shown. Cluster 1 genes have a slightly higher divergence rate than cluster 1 and 2, but genes within all three clusters are relatively equal in their rate of change.

The *IFNT *and two *IFNW *pseudogenes are neighbors at the distal end of sub-locus 1, suggesting that this cluster of genes originated from an *IFNW *that had become isolated from other *IFNW *before the divergence of the *IFNT*. Its unique position outside the *IFNA/IFNW *clusters and close to the edge of the sub-locus may have permitted the rapid expansion and evolution of the *IFNT *family without the restraints placed on the clustered *IFNW*.

All non-ruminant species examined to date, including mouse, human, cats, dogs, rabbits, and pigs, contain only one *IFNB *[16]. In cattle, this family has clearly expanded and extends from the distal end of *IFNA/IFNW *cluster 3 to the end of sub-locus 2. Interspersed within these multiple *IFNB *are members of the previously unidentified *IFN *family, *IFNX*. Again, it is tempting to hypothesize that the *IFNX *and expanded *IFNB *family were able to emerge due to their location on the edge of the sub-locus 2, as suggested for the *IFNT *in sub-locus 1.

### Repetitive Elements within Sub-loci

Repetitive elements have been implicated in gene duplication by creating regions predisposed to homologous recombination [58,59] and also in organizing the assembly of enhanceasomes, as recently described for transcription of IFNB [60]. Over one third of the bovine Type I IFN locus consists of interspersed repeats, rather less than the 43% assessed for the murine Type I IFN locus [27]. Repeats are more enriched in sub-locus 1 largely because of the presence of a greater number of long interspersed nucleotide elements (LINE) and long terminal repeats (LTR) (Table 4). LINE2 elements are absent in both sub-loci. Short interspersed nucleotide elements (SINE)s are present in similar proportions, approximately 15%, for both sub-loci. The arrangement of these elements provides no obvious insight into whether the sequences are involved in gene duplication or in controlling transcription.

**Table 4:** Repetitive elements within the bovine Type I IFN sub-loci.

	**Sub-locus 1**	**Sub-locus 2**
**SINEs:**	15.29%	15.68%
Alu/B1	0.00%	0.00%
MIRs	0.51%	0.81%
		
**LINEs:**	17.74%	11.23%
LINE1	10.33%	2.20%
LINE2	0.44%	0.29%
L3/CR1	0.04%	0.00%
RTE	6.93%	8.73%
		
**LTR elements:**	4.01%	2.75%
MaLRs	0.89%	0.98%
ERVL	0.12%	0.03%
ERV classI	0.79%	0.22%
ERV classII	0.00%	0.00%
		
**DNA elements:**	2.36%	2.68%
MER1_type	1.72%	2.45%
MER2_type	0.45%	0.00%
		
**Unclassified:**	0.00%	0.00%
		
**Total interspersed repeats:**	39.40%	32.35%
		
**Small RNA:**	0.24%	0.40%

The percentage of repetitive elements for the Type I IFN sub-loci was calculated in RepeatMasker 3.1.9. The length of nucleotide sequence encompassing the repetitive element divided by the length of the total nucleotide sequence for either sub-locus 1 or sub-locus 2 was used to calculate the percentage shown in each column. Sub-locus 1 has a higher proportion of LINE1 and LTR elements than sub-locus 2 resulting in a higher total number of interspersed repeats within sub-locus.

### Palindromic IFN within IFNA/IFNW clusters 1 and 2

**Figure 3:**
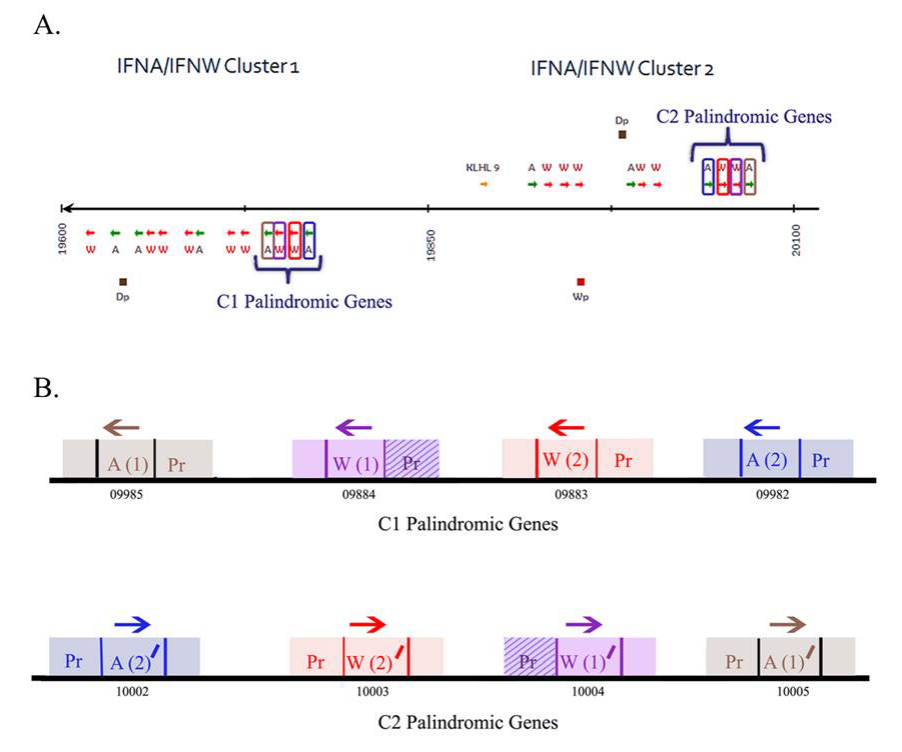
**Palindromic gene sets within IFNA/IFNW clusters 1 and 2**. (A) An enlarged image of *IFNA/IFNW *clusters 1 and 2 from Fig. 2 is shown. (B) The promoter (Pr), ORF, and 3^/ ^UTR for each gene have been depicted in a schematic of both gene sets (not drawn to scale). The direction of gene transcription is indicated by an arrow above each gene and the GLEAN number is written below each gene (GLEAN 09981 [Bovine Genome Database temporary ID:2371], GLEAN 09982 [Bovine Genome Database temporary ID:2258], GLEAN 09983 [Bovine Genome Database temporary ID:2733], GLEAN 09984 [Bovine Genome Database temporary ID:1340], GLEAN 10002 [Bovine Genome Database temporary ID:1155], GLEAN 10003 [Bovine Genome Database temporary ID:2217], GLEAN 10004 [Bovine Genome Database temporary ID:2525], GLEAN 10005 [Bovine Genome Database temporary ID:74]. Genes with 100% nucleotide identity within their promoters, ORFs, and 3^/ ^UTRs are shown in matching solid colors. The only gene pair that does not have 100% nucleotide identity in the promoter, W(1)/W(1)^/^, is indicated by diagonal stripes through the promoter box.

As mentioned previously, a gene set located on the distal end of *IFNA/IFNW *cluster 1 is a palindrome to a gene set on the distal end of cluster 2 (Fig. 3A). Two *IFNA *and two *IFNW *are present in each gene set and these are designated as *A(1) *and *A(2) *for the two *IFNA *and *W(1) *and *W(2) *for the two *IFNW *in cluster 1. *A(1) *and *A(2) *are located at the proximal and distal ends of the gene set in cluster 1, respectively. *W(1) *and *W(2) *are located from proximal to distal between A(1) and A(2). The genes with identical ORFs in cluster 2 are designated as *A(1)*^/^, *A(2)*^/^, *W(1)*^/^, and *W(2)*^/ ^(Fig. 3B). The ORF and the first 350 bp of the 3^/ ^UTR for the two *IFNA *and *IFNW *gene pairs are identical, and an approximately 550 bp promoter for both *IFNA *gene pairs and the *W(2)/W(2)*^/ ^pair are also identical. The promoters of the *W(1)/W(1)*^/^pair, while closely similar (99%), are not identical, however. All nucleotide differences between the *W(1)/W(1)*^/ ^gene pair are within a region between 300 bp and 400 bp upstream of the transcriptional start site.

Two different evolutionary processes, either gene duplication or gene conversion, could possibly explain the existence of the *IFNA/IFNW *palindromic gene sets. Gene duplication involves the formation of a new gene copy. Gene conversion, on the other hand, does not generate new gene copies, but instead homogenizes existing genes. Both gene duplication and gene conversion have been specifically implicated in the evolution of the *IFNA *in human, chimpanzee, dog, rhesus monkey, rat, and mouse [30,61,62]. Gene conversion, specifically, was predicted by two different statistical programs, GARD and GENECONV, in humans, chimpanzee, rhesus monkey, and mice. Furthermore, despite *IFNA *genes aligning in conserved positions on a locus map for chimpanzees and humans, the subfamily separated into species-specific clades on phylogenetic analysis [61], strongly indicating gene conversion has occurred in the *IFNA *subfamily in these two species. Although gene duplications cannot be unambiguously distinguished from gene conversions [63], the latter seldom involve sequence longer than 1 kb in mammals, with 3 kb considered the maximum length [63]. Therefore, when the sequence tract involved is "too large" for gene conversion, gene duplication is usually implicated [64]. The palindomic gene set involves at least a 27 kb tract, far exceeding this size limit and reducing the likelihood of a conversion event. Therefore, a segmental duplication event, which is a specific type of gene duplication that involves a large segment of a locus, combined with an inversion is the best explanation for the palindrome [65].

### Selective Pressure on the ORF of Type I IFN Subfamilies

Comparison of the rate of non-synonymous nucleotide change relative to the rate of synonymous change can provide information about the type of selection operating on the members of a multigene families [66]. If neutral selection is occurring, then all nucleotides in a sequence are equally likely to change. Consequently the rate of synonymous nucleotide changes (dS) will be equal to the rate of non-synonymous changes (dN) and dS:dN will equal 1. Rapid change in the amino acid sequence is the desired endpoint for positive selective pressure. Hence, in this scenario, dN will exceed dS, and dN:dS will be greater than 1. Conversely, if strong selection against amino acid change is present (purifying selection), dN will be less than dS and dN:dS will be less than 1. Virtually all pairwise comparisons within *IFNA*, whatever the species [61,67], and *IFNT *[57] have shown the overall value for dN not to be significantly higher than dS. Indeed, dN values have been generally calculated to be lower than dS, consistent with the conclusion that there has not been strong positive selection for amino acid change within the coding regions of these subfamilies of *IFN*.

**Figure 4:**
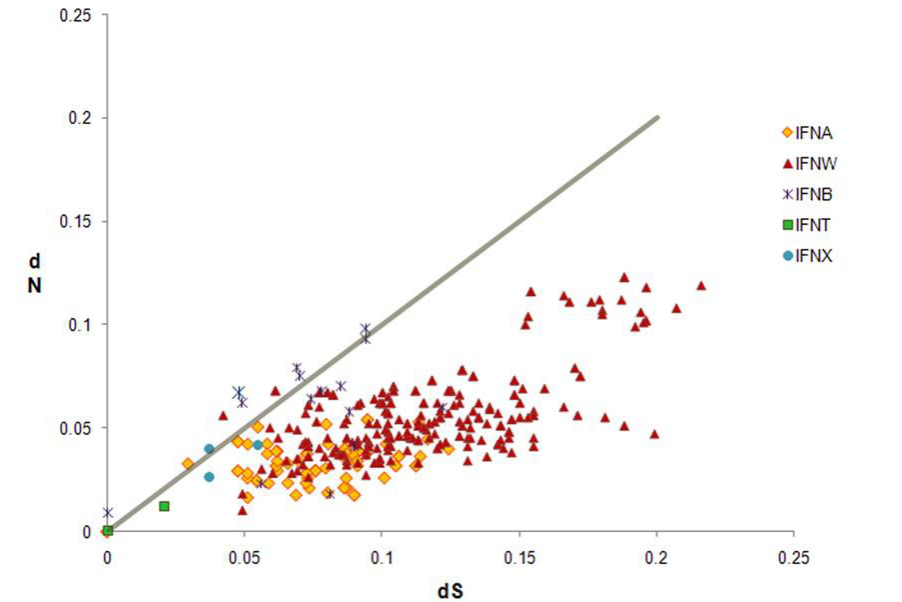
**Selective Pressure on the coding regions of bovine Type I IFN**. Pairwise analysis of the coding region between every gene pair in a Type I IFN subfamily was used to calculate dS and dN, and the two values were plotted against one another. No comparisons were made between genes belonging to different subfamilies, i.e. an IFNB gene and an IFNA gene. In addition, all probable pseudogenes, which contained early stop codons or frameshift mutations, were not included in the analysis. The grey diagonal line in the graph represent neutral selection rate where dS = dN. Gene pairs undergoing positive selection would appear above the diagonal and gene pairs undergoing purifying selection would appear below the diagonal. The graph clearly shows that no positive selection is occurring within the coding regions of any Type I IFN family. Furthermore, *IFNW *and *IFNA *subfamilies appear to be undergoing purifying selection.

The dN:dS for all multigene bovine *IFN *subfamilies, including only *IFN *annotated during this work, is illustrated in Fig. 4. The dN and dS values for every gene pair in a Type I IFN subfamily were calculated and plotted against each other. No multigene IFN subfamily (*IFNA*, *IFNB*, *IFNW*, or *IFNT*) in bovine has a dN significantly exceeding dS. In fact, bovine *IFNA *and *IFNW *provide strong evidence for purifying selection. The significance of purifying selection within *IFNW *and *IFNA *subfamilies was also verified through a codon based Z-test (p < 0.001), which determines selective pressure for a gene pair or group of genes based on the difference between dN and dS through a one-tailed t-test (Table 5). Four *IFNB *pairs out of 15 pairwise comparisons examined provide some evidence for purifying selection on the basis of the pairwise codon based Z-test (Table 6), but such selective pressures are not evident for the family as a whole (p = 0.171). On the other hand, the same test employed for neutral selection provided no evidence for a lack of selective pressure operating on any members of the *IFNB *subfamily (p = 0.343), indicating the six genes comprising the bovine *IFNB *subfamily may be too few for a meaningful analysis. Since only three *IFNT *were identified, statistical analysis of this subfamily from the genomic data was not possible.

**Table 5:** Purifying selection within IFNW and IFNA coding regions.

**Family**	**p-Value**	**dS-dN**
IFNA	0.0	3.824
IFNW	0.0	4.013

The significance of purifying selection in the coding regions of *IFNW *and *IFNA *subfamilies was verified through a codon based Z-test analyzing the overall average for the subfamilies. The p-value is shown in the second column with p-values less than 0.05 considered significant. The test statistic, the difference between dN and dS, is shown in the third column. The variance of the difference was computed by using the bootstrap method (1000 replicates).

**Table 6:** Selection in IFNB.

	**24309**	**24311**	**24313**	**24315**	**24317**	**24318**
**IFNB (24309)**						
**IFNB (24311)**	0.486					
**IFNB (24313)**	1.000	1.000				
**IFNB (24315)**	0.032	0.339	0.342			
**IFNB (24317)**	0.303	1.000	1.000	0.036		
**IFNB (24318)**	0.150	1.000	1.000	0.008	0.050	

Pairwise comparisons between *IFNB *through the codon based Z-test are shown in the above table with the statistically significant comparisons underlined.

The classic model of gene duplication states that after a duplication event one gene continues to perform the ancestral function while the second either rapidly evolves to fill a new niche or becomes inactive [65,68,69]. As a consequence gene duplication is usually followed by a period in which there is an acquisition of non-synonymous nucleotide changes in one of the two genes, leading to a divergence in amino acid sequence. This temporary relaxation of purifying selection, in which dN:dS approaches 1, permits the gene to become fine-tuned to its new role or, more commonly, results in pseudogenization. Such a sequence of events does not appear to have occurred during the large scale expansion of the *IFNW *family where there is strong evidence for purifying selection operating on the coding regions of the genes (Fig. 4 & Table 5). One potential explanation is that sub-functionalization is occurring through alterations in the manner in which these genes are subjected to transcriptional control rather than in the structure of the proteins themselves. Evidence for positive selective pressure in the regulatory regions of IFNW would strongly support this hypothesis, but a detailed promoter analysis is beyond the scope of this work.

### IFNX: Evidence for a novel subfamily

**Figure 5:**
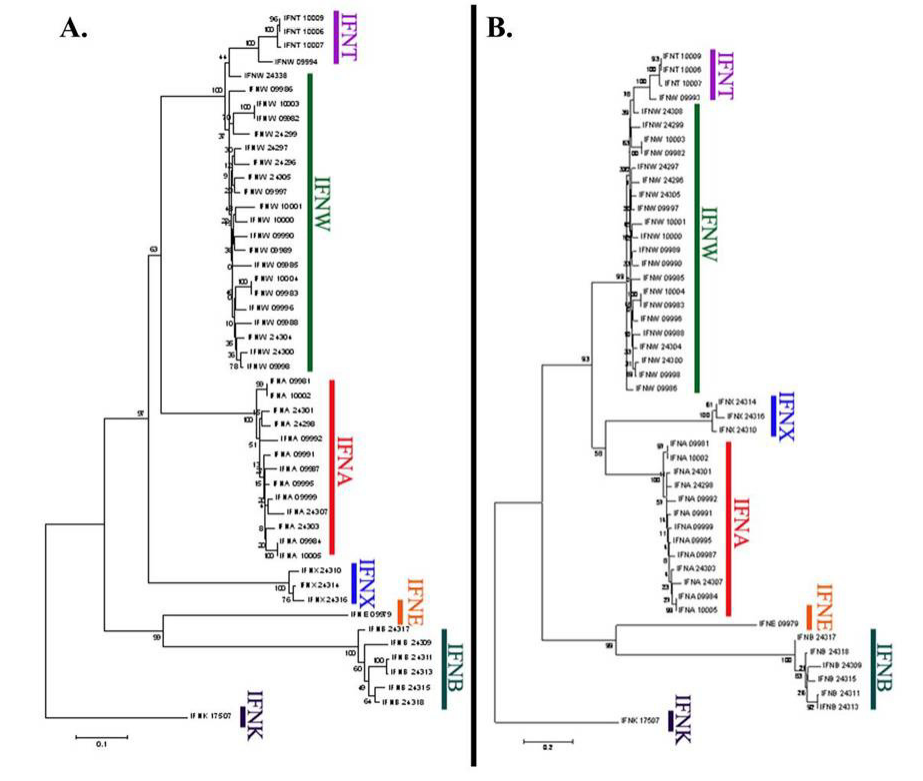
**The Bovine Type I IFN Phylogenetic Tree**. (A) The evolutionary history of bovine Type I *IFN *was inferred by using the Neighbor-Joining (NJ) method with bootstrap test (1000 replicates). The tree was rooted to *IFNK *and calculations were based on uniform rates of change for all sites. *IFNX *emerged prior to *IFNA *in this analysis. (B) The tree illustrates the evolutionary history of bovine *IFN *based on different rates of change between sites (gamma = 1). The tree was again based on the NJ method with bootstrap test (1000 replicates) and rooted to *IFNK*. *IFNX *and *IFNA *branched from a common ancestor in this analysis.

Based on the current assembly, the IFNX subfamily consists of three potential genes and one probable pseudogene. The origin of these genes is currently unclear, but they appear to constitute a unique IFN subfamily, whose closest relatives are the *IFNA *based on a Maximum Composite Likelihood divergence analysis, with *IFNX *sharing over 65% nucleotide identity to *IFNA *and only about 45% nucleotide identity to *IFNB*. They are clearly defined on phylogenetic trees based on their ORF as a distinct clade that is most closely related to *IFNA*; however, phylogenetic analyses are conflicting with regard to when *IFNX *first appeared. Phylogenetic trees calculated on the assumption that all nucleotide sites within the coding sequence change at the same rate indicate *IFNA *separated from *IFNX *prior to or corresponding with the radiation of eutherian mammals, i.e. more than 150 MYA [35] (Fig. 5A). Conversely, phylogenetic trees that take into account substitution rate variation from site to site indicate that bovine *IFNX *and bovine *IFNA *emerged from a common ancestor after the radiation of the major mammalian orders (Fig. 5B). In other words, this model recognizes that certain amino acids and, by corollary certain nucleotides, are more highly conserved than others across subfamilies. This second model most closely matches the amino acid differences among IFN subfamilies, since amino acids critical to preserving the tertiary structures of IFNB, IFNA, IFNT, and IFNL are much more highly conserved than ones in less critical regions of the proteins [55,70]. Preliminary examination of IFNX indicate some of the more highly conserved amino acids for other Type I IFN, such as cysteine residues 1 and 99 (discussed below), are also conserved in IFNX. In addition *IFNX *and the *IFNX *pseudogene are absent in human, mouse, porcine, feline, and canine genomic databases, yet some remnants of their presence might be expected if this family emerged early in the evolution of mammals and prior to the main radiation events.

**Figure 6:**
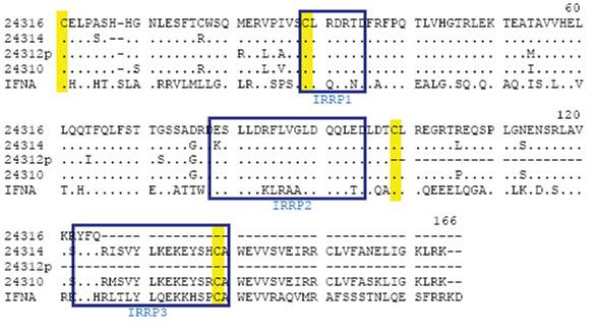
**IFNX alignment**. The coding region, minus the predicted signal peptide, for the three predicted IFNX genes are denoted by their GLEAN numbers (24314 [Bovine Genome Database temporary ID:2633], 24310 [Bovine Genome Database temporary ID:2546], and 24316 [Bovine Genome Database temporary ID:2733]) and are aligned with bovine *IFNA *[Genbank:DQ396807]. The mature coding sequence of the three potentially expressed *IFNX *genes differs as follows 164 amino acids (aa) for Glean 24310, 163 aa for Glean 24314, and 124 aa for 24316. Conserved cysteine residues form disulfide bonds in IFNA between positions 1→99 and 29→139 and are shown in yellow. Glean 24316 has an early stop codon that eliminates the second disulfide bond, but both Glean 24314 and Glean 24310 encode these conserved residues. Regions strongly associated with receptor binding in IFNA, or interferon receptor recognition peptides (IRRP), are boxed in blue.

Two of the three potential *IFNX *genes contain the four conserved cysteine residues required for the disulfide bonds (1–99; 29–139) encountered in *IFNA *and those Type I subfamilies that emerged from *IFNA*, namely *IFNW*, *IFND*, and *IFNT*. The third potential gene, GLEAN 24316 [Bovine Genome Database temporary ID:2755], contains an early termination codon at codon 125, which eliminates the second disulfide bond (29–139) (Fig. 6), raising the possibility that it is a second pseudogene. The "trace-WGS" database was visually examined to verify the presence of this early stop codon. Five BACs, all of which were in the minus orientation, contained sequences that exactly matched the complementary sequence to Glean 24316, i.e. all contained the early termination codon.

Previous studies of IFNA have identified three regions that are strongly associated with IFN-receptor interaction and are termed interferon receptor recognition peptides (IRRP)1–3 [70-72]. IRRP1 (27–35) and IRRP2 (78–105) control the initial binding of IFN to the Type I receptor and are highly conserved among IFNA. IRRP3 (123–140) modulates the downstream signaling pathways, so that amino acid changes in this region can explain some differences in biological activity among different IFNA. The protein products of the three *IFNX *do not possess identical IRRP1 and IRRP2 motifs as IFNA, but these two regions are highly conserved within the subfamily, emphasizing, first, the possible importance of this motif and second that the IFNX family is unique and distinct from IFNA. IRRP3 was absent in GLEAN 24316 again suggesting that it may be a pseudogene. The two remaining IFNX members differed in their IRRP3 sequences, a not unexpected finding as changes in this region may provide subtle differences in biologic activity between the two family members. None of the IFNX genes contain the N-glycosylation sequence (N-X-S/T) common in other Type I IFNs that could alter IFN-receptor interaction.

No evidence for *IFNX *expression could be found in any EST databases, although, genes with high identity to *IFNX *exist in the equine genomic database. The conservation of this gene family in species that diverged at least 80 million years ago suggests that the family may have an important function in ungulates. However, the apparent absence of *IFNX *genes in pigs, also an ungulate, is puzzling. Possibly, *IFNX *has a specific function in herbivores that is not required in omnivores, most likely in immune defense against particular viruses or other pathogenic organisms affecting such species.

The identification of a novel Type I IFN gene, the *IFNX*, is an unexpected and possibly important finding. The proteins encoded by this family of genes differ sufficiently in primary sequence from related Type I IFN to justify a separate designation from the related IFNA and IFNB. The presence of a distinct cluster of *IFNX *within the Type I IFN locus, the phylogenetic position of *IFNX *as a separate clade within the IFN tree, and the conservation of critical amino acid residues, are totally consistent with classifying the *IFNX *as a distinct Type I IFN subfamily. Whether IFNX are responsive to a viral challenge and able to interact with the Type I IFN receptor and elicit a typical Type I response in their target cells has yet to be verified. Substantial work will be necessary to characterize this subfamily fully, but its place as a separate clade within the Type I IFN would appear to be assured.

## Conclusion

The Type I *IFN *locus has undergone substantial transformation in ruminants compared to humans and mice. The conserved locus structure has been transformed, subfamilies have expanded, and two subfamilies not present in either humans or mice exist. The division of the locus into two sub-loci may have provided an opportunity for genes to duplicate and contribute to an expanded function of the Type I IFN. The divergence of the successful pecoran ruminant sub-order and its geographic spread might have required improved protection against unique ruminant pathogens. The *IFNX *sub-family and the greatly expanded ruminant specific *IFNW *are likely candidates for providing such protection. Radically new functions for Type I IFN might also have been gained, such as the one exemplified by the *IFNT*, whose appearance coincided with, and possibly permitted, the acquisition of the unique, synepitheliochorial placentation that characterizes the Ruminantia sub-order and requires powerful conceptus signaling before the trophoblast has even attached to the uterine wall [24]. The ancient Type III IFN (*IFNL/IL28-29*) may have become a casualty of the expansion and broadened the role of the Type I locus, as only an inactive *IFNL *remains in the bovine genome. It is tempting to speculate that the function of *IFNL *has been replaced as the component genes of the Type I *IFN *locus expanded.

The authors concede that the bovine genome assembly is a work in progress and that the predicted arrangement of individual *IFN *genes may have to be modified as data are reanalyzed. In addition, it is clear that an individual animal possesses unique genomic peculiarities, including inversions, duplications, and presence and absence of specific genes and that the IFN locus of a single Hereford cow may not be replicated precisely in other breeds. Nevertheless, with the exception of the size of the "gap" between the two sub-loci, the organization and sequence of the bovine Type I IFN have remained relatively constant through the most recent assemblies. The unique features of the locus, which include the presence of the gap itself, the arrangements of *IFNW/IFNA *clusters, the dramatic expansion of the *IFNW*, the presence of the *IFNX*, and the separation of *IFNT *from the *IFNW/IFNA *clusters are consistent observations and unlikely to undergo drastic re-evaluation in future versions of the assembly.

## Authors' contributions

AMW designed the study and performed all bioinformatics. RMR proposed and supervised the study. Both authors wrote, read and approved the manuscript.
